# Mariana serpentinite mud volcanism exhumes subducted seamount materials: implications for the origin of life

**DOI:** 10.1098/rsta.2018.0425

**Published:** 2020-01-06

**Authors:** Patricia Fryer, C. Geoffrey Wheat, Trevor Williams, Christopher Kelley, Kevin Johnson, Jeffrey Ryan, Walter Kurz, John Shervais, Elmar Albers, Barbara Bekins, Baptiste Debret, Jianghong Deng, Yanhui Dong, Philip Eickenbusch, Emanuelle Frery, Yuji Ichiyama, Raymond Johnston, Richard Kevorkian, Vitor Magalhaes, Simone Mantovanelli, Walter Menapace, Catriona Menzies, Katsuyoshi Michibayashi, Craig Moyer, Kelli Mullane, Jung-Woo Park, Roy Price, Olivier Sissmann, Shino Suzuki, Ken Takai, Bastien Walter, Rui Zhang, Diva Amon, Deborah Glickson, Shirley Pomponi

**Affiliations:** 1 School of Ocean and Earth Science and Technology, University of Hawaii at Manoa, Honolulu, HI, USA; 2 University of Alaska, Fairbanks, AK, USA; 3 International Ocean Discovery Program, Texas A&M University, College Station, TX, USA; 4 School of Geosciences, University of South Florida, Tampa, FL, USA; 5 Institute of Earth Sciences, University of Graz, NAWI Graz Geocenter, Institute of Earth Sciences, Graz, Austria; 6 Department of Geology, Utah State University, Logan, UT, USA; 7 Department of Geosciences, University of Bremen, Bremen, Germany; 8 United States Geological Survey, NASA Ames, Mountain View, CA, USA; 9 University of Cambridge, Cambridge, UK; 10 School of Earth and Space Sciences, University of Science and Technology of China, Hefei, Anhui Province, People's Republic of China; 11 Key Laboratory of Submarine Geoscience, Second Institute of Oceanography, State Oceanic Administration, Hangzhou, Zhejiang Province, People's Republic of China; 12 Department of Environmental Systems Science, ETH Zurich, Zurich, Switzerland; 13 Commonwealth Scientific and Industrial Research Organisation, Kensington, Western Australia, Australia; 14 Department of Earth Sciences, Chiba University, Chiba, Chiba Prefecture, Japan; 15 Department of Microbiology, University of Tennessee, Knoxville, TN, USA; 16 Por Portuguese Institute for Sea and Atmosphere (IPMA), Rua C ao Aeroporto, Lisbon, Portugal; 17 Oceanographic Institute, São Paulo University, São Paulo, Brazil; 18 MARUM - Center for Marine Environmental Sciences, Department of Geosciences, University of Bremen, Bremen, Germany; 19 Ocean and Earth Science, National Oceanography Centre, University of Southampton, Southampton SO14 3ZH, UK; 20 Department of Earth and Planetary Sciences, Graduate School of Environmental Studies, Nagoya University, Nagoya, Aichi Prefecture, Japan; 21 Biology Department, Western Washington University, Bellingham, WA, USA; 22 Scripps Institution of Oceanography, University of California, San Diego, CA, USA; 23 School of Earth and Environmental Sciences & Research Institute of Oceanography, Seoul National University, Gwanak-gu, Seoul, Republic of Korea; 24 School of Marine and Atmospheric Sciences, State University of New York, Stony Brook, NY, USA; 25 IFP Energies Nouvelles, 92500 Rueil-Malmaison, Ile-de-France, France; 26 Kochi Institute for Core Sample Research, Japan Agency for Marine-Earth Science and Technology (JAMSTEC), Nankoku, Kochi Prefecture, Japan; 27 Department of Subsurface Geobiological Analysis and Research (D-SUGAR), Japan Agency for Marine-Earth Science and Technology (JAMSTEC), Natsushima-cho, Yokosuka, Kanagawa Prefecture, Japan; 28 GeoResources, Universite de Lorraine, Vandoeuvre-les-Nancy, Cedex, France; 29 State Key Laboratory of Marine Environmental Sciences, Institute of Marine Microbes and Exospheres, Xiamen University, Xiang'an Campus, Xiamen, Fujian Province, People's Republic of China; 30 Life Sciences Department, Natural History Museum, London, Cromwell Road, London, UK; 31 Board on Earth Sciences and Resources, National Academies of Sciences, Engineering, and Medicine, Washington, DC, USA; 32 NOAA Cooperative Institute for Ocean Exploration, Research, and Technology, Harbor Branch Oceanographic Institute, Florida Atlantic University, Fort Pierce, FL, USA

**Keywords:** Mariana trench, serpentinite mud volcanism, subducted cretaceous seamounts, exhumed microbes, evolution of life

## Abstract

The subduction of seamounts and ridge features at convergent plate boundaries plays an important role in the deformation of the overriding plate and influences geochemical cycling and associated biological processes. Active serpentinization of forearc mantle and serpentinite mud volcanism on the Mariana forearc (between the trench and active volcanic arc) provides windows on subduction processes.  Here, we present (1) the first observation of an extensive exposure of an undeformed Cretaceous seamount currently being subducted at the Mariana Trench inner slope; (2) vertical deformation of the forearc region related to subduction of Pacific Plate seamounts and thickened crust; (3) recovered Ocean Drilling Program and International Ocean Discovery Program cores of serpentinite mudflows that confirm exhumation of various Pacific Plate lithologies, including subducted reef limestone; (4) petrologic, geochemical and paleontological data from the cores that show that Pacific Plate seamount exhumation covers greater spatial and temporal extents; (5) the inference that microbial communities associated with serpentinite mud volcanism may also be exhumed from the subducted plate seafloor and/or seamounts; and (6) the implications for effects of these processes with regard to evolution of life.

This article is part of a discussion meeting issue ‘Serpentine in the Earth system’.

## 1. Introduction

### (a) Serpentinization at the Mariana convergent plate margin

Recent (2016) expeditions to the western Pacific's Mariana Trench region, on the United States' National Ocean and Atmospheric Administration's Office of Exploration and Research (NOAA OER) ‘Deepwater Exploration of the Marianas' [1,2] and the International Ocean Discovery Program (IODP) Expedition 366 [3], provided a deeper understanding of the potential for widespread recycling of components of the subducting oceanic lithospheric plate and of seamounts on it. It has long been known that serpentinization is widespread within the Mariana suprasubduction-zone shallow (0 to approx. 19 km) mantle wedge [4,5]. Vertical tectonic deformation creates deep faulting in the forearc region, between the trench and active volcanic arc. These faults result from the increasing curvature of the forearc with time, the rollback eastward of the Pacific Plate and the subduction of seamounts on the latter. The faults permit fluids originating from the subduction channel to permeate the forearc mantle and erupt to form immense (up to 50 km diameter and 2.5 km high) serpentinite mud volcanoes [4,5]. All of the mud volcanoes sampled by deep ocean drilling so far have recovered fragments of subducted plate features.

Serpentinization occurs by the hydration of mantle minerals through well-known reactions, e.g. olivine plus silica in solution will react to produce serpentine; in the absence of excess silica, olivine reacts to form serpentine plus brucite (Mg(0H)_2_) (e.g. [6,7]). The serpentinization of enstatite to form bastite releases excess silica, so hydration of enstatite-poor refractory peridotite tends to favour brucite formation (e.g. [6]).

The serpentinization of mantle peridotites by seawater commonly leads to the oxidation of Fe^2+^-bearing phases (i.e. olivine) to form Fe^3+^-bearing phases, such as magnetite at high temperature or Fe^3+^-serpentine at low temperature [7,8], the mass balance necessarily involves the liberation of hydrogen

$$2\text{FeO}+{\text{H}}_{2}\text{O}(\text{aq})=\text{F}{\text{e}}_{2}{\text{O}}_{3}+{\text{H}}_{2}(\text{aq}).$$

Given the large range of temperatures (from less than 120°C to about 450°C, based on oxygen isotopes) recorded by forearc serpentinites recovered during IODP Expedition 366 [9] and in previous expeditions [10–12], and the diversity of Fe-bearing phases observed in these rocks (e.g. hydroandradite, lizardite, antigorite, magnetite, chromite, brucite), the reaction pathway of H_2_ production in the forearc is likely to be multiple and influenced by many parameters such as the composition and redox state of slab derived fluids.

Indeed, for example, if carbon dioxide is present in the hydrating fluid, serpentinization can also release methane

$$18\text{ M}{\text{g}}_{2}\text{Si}{\text{O}}_{4}+6\text{ F}{\text{e}}_{2}\text{Si}{\text{O}}_{4}+26\;{\text{H}}_{2}\text{O}+\text{C}{\text{O}}_{2}=12\text{ M}{\text{g}}_{3}\text{S}{\text{i}}_{2}{\text{O}}_{5}{\left(\text{OH}\right)}_{4}+4\text{ F}{\text{e}}_{3}{\text{O}}_{4}+\text{C}{\text{H}}_{4},$$

and, if Fe-Ni alloys are present, more complex hydrocarbons [13]. The hydrocarbons released from these reactions are important for sustaining metabolic processes of microbial communities found in the mudflows from the Mariana serpentinite mud volcanoes [4,5,14,15].

Our recent findings suggest that the recycling of subduction channel constituents may include microbial communities from the subducting plate that survive sterilization because of possible temperature variations within a high-relief subduction channel. Towards the end of this paper, we present arguments discussing the environment of serpentinization within the suprasubduction-zone mantle and its potential as a locale for the early Earth's development of life.

### (b) Geologic setting

**Figure 1.**
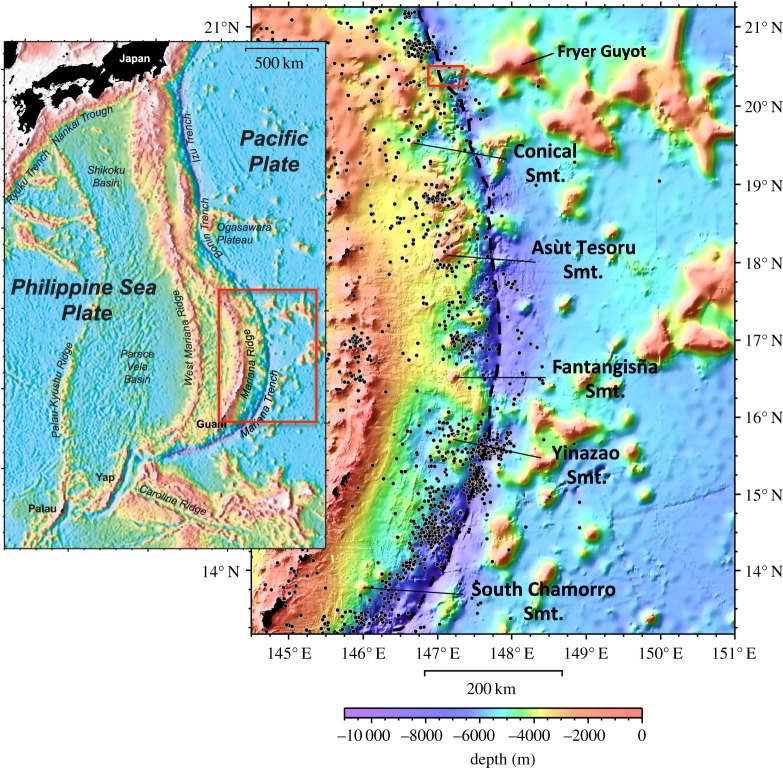
Colour-contoured bathymetry map of Mariana forearc area (inset: regional map) lit from northwest. Forearc serpentinite mud volcanoes drilled to date are labelled. Conical Smt. (ODP Leg 125—Sites 778, 779 and 780); Asùt Tesoru Smt. (IODP Exp. 366 - Sites U1493, U1494, U1495, U1496); Fantangisña Smt. (IODP Exp. 366—Sites U1497, U1498); Yinazao Smt. (IODP Exp. 366—Sites U1491, U1492); and South Chamorro Smt. (ODP Leg 195—Site 1200). Black dots are positions of all earthquakes from 1900 to 2017 at depths of 0–50 km (NEIC database). Black dashed line is Mariana Trench axis. Large box on map at left is area of map on right. Small red box at 12.5°N, 147°E indicates location of figure 2*a*. (Map created by N.C. Becker). (Online version in colour.)

The western Pacific seafloor, east of the Mariana Trench, has hundreds of seamounts close to the trench [13] (figure 1). As the Pacific lithospheric plate and features on it approach the trench, lithospheric bending creates an outer trench bulge about 60–120 km east of the trench. This decreases the effective elastic plate thickness [16–18] so that normal faulting creates uplifted blocks with intervening troughs and/or trenchward, down-stepping half-grabens. These features are generally oriented parallel to the trench axis [19]. Small seamounts on the subducting plate are strongly affected by outer-rise deformation, but large ones remain essentially intact [20].

Impinging plate seamounts make first contact with exposed mantle at the inner slope of the Mariana Trench, which is the ‘type’ non-accretionary convergent plate margin [21]. Here, little or no sediment accumulates at the leading edge of the overriding plate and the suprasubduction-zone mantle wedge is exposed below approximately 6 km on the inner trench slope. Igneous and sedimentary rocks of the forearc are exposed at shallower depths, sparsely capped by sediments. Uplift of the forearc in addition to along- and across-strike extension has created faults on many scales over the approximately 50 Myr history of convergence [22,23]. Deep faults, penetrating to the subduction channel [20], provide pathways for fluids from the down-going plate to hydrate and serpentinize the forearc mantle [4,5,24–26]. Comminuted fault gouge, mobilized by the ascending fluids, creates a mush that rises through fault-controlled conduits to the sea floor, and debouches to form huge serpentinite mud volcanoes within about 100 km west of the trench [4,5]. The Mariana forearc serpentinite mud volcanoes episodically [3,4] tap the subduction channel at a range of depths and temperatures (table 1), providing windows into aspects of physical, chemical and biological processes that affect the subducted Pacific Plate as it moves downward and the overlying forearc lithosphere [5].

**Table 1.** Physical and chemical characteristics of summits of drilled serpentinite mud volcanoes in relation to the Mariana Trench.

measurement	Yinazao Seamount	Fantangisña Seamount	Asùt Tesoru Seamount	South Chamorro Seamount	Conical Seamount
distance to trench (km)	55	62	72	78	86
depth to slab (km)	13	14	18	18	19
temperature of slab (°C)	∼80	∼150	∼250	250–350	250–350
pore water pH	11.2	11	12.5	12.5	12.5
pore water Ca (mM)	64	90	0.1	0.3	1
pore water K (mM)	∼1	5	14	19	15

## 2. New observation of seamount subduction

In June 2016, we recorded the first observation of a large undeformed seamount that is exposed in the inner slope of the Mariana Trench. The only previous sighting of Cretaceous reefal material from a subducted seamount was in the late 1980s on a series of Nautile submersible dives in the deepest 20 km of the western inner slope of the Japan Trench [27]. This was adjacent to the impinging Pacific Plate's Daiichi Kashima Seamount. These observations were consistent with the suggestion that because of the strong deformation of subducting plates proximal to trenches, and because of loss of buoyancy, subducting Pacific Plate seamounts will be rotated and sheared as they enter a subduction channel [28]. This is not at all what we observed on the deep inner slope of the Mariana Trench.

**Figure 2.**
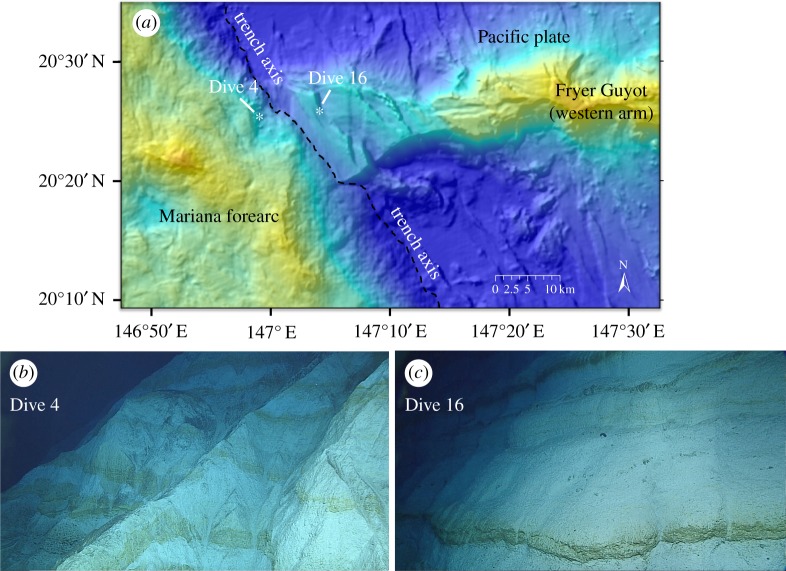
(*a*) Colour-contoured bathymetry map of Mariana Trench area (small red box at ∼12.5°N, 147°E in figure 1), lit from northwest (cool/dark colours are deep, warm/light colours are shallower). White asterisks show positions of ROV Dives 4 [1] and 16 [1] at approximately 20°N. Black dashed line is Mariana Trench axis. (*b*) ROV video grab of oblique view of reef sequences on ROV Dive 4 on the deep inner slope of Mariana Trench (light layers are massive reef deposits (rudists, figure 3), darker layers are mainly bivalve fossils). Debris chutes and intervening sharp ridges are from submarine mass-wasting. (*c*) ROV video grab of oblique view on ROV Dive 16 of Cretaceous reef sequences on Pacific Plate Cretaceous guyot, light layers are massive reef deposits and rudists ( figure 3 for scale) and darker layers are mainly bivalve fossils. (Online version in colour.)

**Figure 3.**
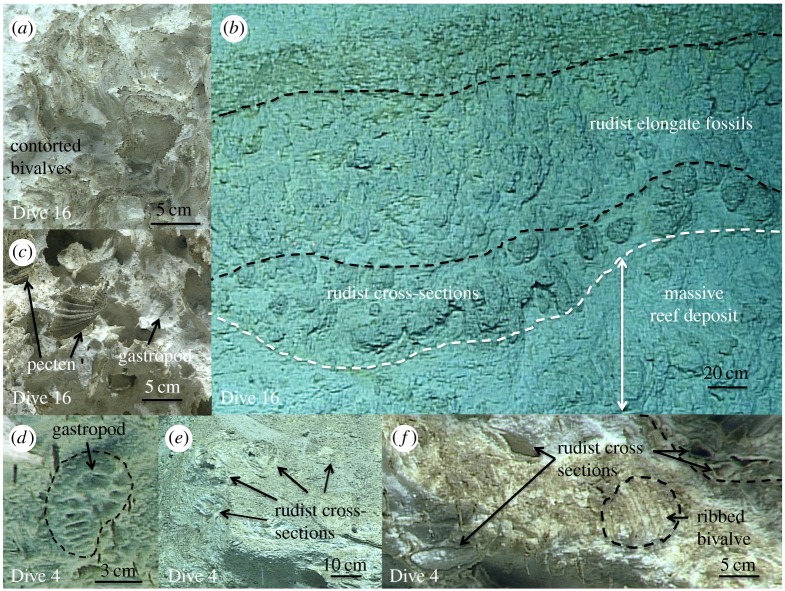
(*a*–*c*) Video grabs of fossils observed on ROV video on Fryer Guyot (Dive 16 [1]). (*d*–*f*) Video grabs of fossils observed on ROV video on Mariana Trench inner slope (Dive 4 [1]). (Fossil identifications verified by S. Stanley, personal communication, 2017).

We used the remotely operated vehicle (ROV) Deep Discoverer during the R/V Okeanos Explorer's Leg 3 of the National Oceanographic and Atmospheric Administration's (NOAA), Office of Exploration and Research (OER) 2016 ‘Deepwater Exploration of the Marianas' cruise [1,2]. On Dive 4 (at approx. 20.5° N) [1], we observed a 275-m-high sequence of exposed reef, starting at 5 995 m (figure 2*a*). These reef sequences are morphologically identical to those we observed on Dive 16 [2], during which we drove the ROV up a summit escarpment of Fryer Guyot (approx. 20.5° N), a Cretaceous-aged, flat-topped seamount immediately east of the trench (figure 2*b,c*). We interpret the Dive 4 exposure to be part of a Cretaceous reef complex of a seamount that is partially subducted beneath the edge of the overriding Mariana forearc. The reefal sequences on Dive 16 show numerous *in situ* fossils (figures 3*a–c*) including bivalves. White layers that have a rudist morphology (S. Stanley, 2017, personal communication) alternate with tan bands of other reef fossils (figure 3*a–c*). Similar sequences occur on Dive 4 (figure 3*d*–*f*). Slump scars observed on Dive 4 indicate mass wasting, but there is no indication of shearing, rotation or any large-scale deformation. The ROV left the bottom at a depth of about 5 720 m on the inner trench slope to perform a mid-water survey, but the traverse of the subducting seamount ended without seeing the upper margin of the reef exposure.

## 3. Mariana forearc deformation and mud volcano distribution

In general, contact with the overriding plate edge at the initial subduction of high-relief features leads to uplift and erosion of the overriding plate and formation of adjacent deep basins or grabens [29–31]. Bathymetric relief in the Mariana forearc is significant within about 100 km of the trench [32]. Several broad ridges, shallower than the average regional depth along strike, cross the entire forearc (figure 1). They lie collinear with the convergence direction of thicker Pacific Plate crust [33] and with clusters of Pacific seamounts (figure 1). Similar morphologic effects occur on other convergent margins where subduction of ridges drive thickening of forearc crust that is commensurate in dimensions with that of the impinging ridge (e.g. Cocos Ridge [34]). The uplifted Mariana forearc from 19.5° N to 21° N (figure 1) is shallower by 2–3 km than the general 4 km deep bathymetry along strike at about 100 km west of the trench axis. Both north and south of this ridge, the seafloor has deep basins (greater than 5 km) [35]. There are narrower ridges that extend across the forearc: one from approximately 17.5° to 19° N; one from approximately 16° to 16.75° N; and an outer forearc ridge from approximately 15.25° to 15.5° N. All of these are bounded by anomalously deep seafloor (figure 1).

Faulting related to vertical tectonic deformation, to east–west extension caused by the Pacific Plate's eastward roll-back from the trench, and to north–south extension caused by the forearc's increase in curvature with time, is extensive and distribution of serpentinite mud volcanism on the Mariana forearc is related to fault lineaments [4,35–37]. These serpentinite mud volcanoes erupt episodically over millions of years [35]. It is likely their eruptions are triggered by release of slab-derived fluids in association with seismic events [4,5,36,37]. The mudflows are composed largely (greater than 90%) of clay- to sand-sized serpentine grains with pebbles and larger clasts of variably serpentinized forearc mantle and forearc crustal rocks [4,5,25].

## 4. Forearc mantle serpentinization

The potential degree of serpentinization of the Mariana forearc mantle by slab-derived fluids has been suggested to reach 100% over the approximately 50 Myr history of subduction at the Mariana convergent margin system [38]. However, dredging, coring and submersible/ROV recovery of peridotites from the forearc show that many are only partially serpentinized [22]. Thus it is likely that the greatest degree of serpentinization is constrained to fracture-related pathways (e.g. [25,26]). The results of Ocean Drilling Program (ODP) coring of serpentinite mud volcanoes are consistent with this interpretation.

Ultramafic rocks from Conical Seamount (figure 1) recovered in dredging and ODP Leg 125 [39–42] coring show variability in the degree of serpentinization from about 40% to 100%. Most of the ultramafic rock clasts are harzburgite and subordinate dunite, both of which show evidence of melt extraction in a suprasubduction-zone environment [40]. Primary modal mineralogy of the Conical Seamount harzburgites includes olivine (75–95%); orthopyroxene (2–25%); clinopyroxene (1–3%) and spinel (greater than 1%) [39]. Primary modal mineralogy of dunites consists of olivine (90–99%), orthopyroxene (1–9%) and up to 1% spinel [42]. Both dredged rocks [39] and drill core samples [40–42] have essentially the same range of degree of serpentinization and variability in texture. Olivine generally forms a massive mesh-textured fabric and relict bastitic orthopyroxene sometimes shows kink-banding indicative of induced stress [42,43]. Some harzburgite clasts contain millimetre-sized interstitial clinopyroxene and most have chrome-spinel [42,43]. The serpentine phases present include antigorite (often pseudomorphically replaced by lizardite) and veins containing chrysotile. These may be anywhere from sub-millimetre veinlets to veins tens of millimetres wide. Dunite clasts are generally the most highly serpentinized, with lizardite in mesh texture, occasional remnant antigorite splays and secondary veins of chrysotile [40].

Drill cores on Conical Seamount included both summit and southeast flank sites and there were subtle systematic variations between them [42]. Of particular interest is the variation in degree of serpentinization in flank Site 779, which generally decreases down hole to a depth of approximately 125 m below sea floor (mbsf), but increases from approximately 170 mbsf to 235 mbsf. These types of variation are suggestive of differences in source regions for the mudflows, and analysis with X-ray diffraction of core samples from Hole 779A indicates at least six separate mudflow units [41]. The ultramafic clasts at the summit hole, which was drilled into the conduit of the mud volcano, are more highly serpentinized (61% to 100%) harzburgite, and have nearly uniform composition, but have an increase in degree of serpentinization and alteration with depth [42], suggesting a slow but continuous rate of percolation of pore fluids within the conduit. Very slow fluid emanation from carbonate chimney structures was noted in Alvin dives on Conical Seamount's summit [22].

The summit of South Chamorro Seamount (figure 1) was drilled at Site 1200 on ODP Leg 195 [43]. The ultramafic fraction of the rock clasts recovered in the serpentinite mudflow matrix was dominated by harzburgite with minor dunite. There was a single fragment of lherzolite. The degree of serpentinization (40% to 100%) of these rocks at Site 1200 and the range in textures were essentially similar to those observed on Conical Seamount, although there was little diversity in degree or type of serpentinization at Site 1200 [43]. All the Site 1200 drill holes were confined to a small region at the summit of the mud volcano in order to locate an optimal site for a cased hole. Post-cruise analyses of the rock clasts and mudflow matrix material indicated that the matrix was formed from the clasts by diminution within the conduit [44]. The matrix material reflects a dominant ultramafic composition with about a 10% addition of a subducted plate component [44].

There is little compositional difference, in general, between the Leg 195 ultramafic clasts versus those from Leg 125 with regard to Al_2_O_3_, Fe_2_O_3_, MnO and Na_2_O abundances, although the Leg 195 ultramafics have slightly higher loss on ignition (LOI), MgO, Cr and Ni [45]. The ultramafic rocks from both sites are interpreted to derive from the suprasubduction-zone mantle wedge and have experienced melt extraction at up to 25% to 30% [42–46].

## 5. Metabasites in serpentinite mudflows

Both serpentinite mud volcanoes, Conical and South Chamorro Seamounts, previously dredged, cored, and dived on with submersibles and ROVs, also yielded rocks from forearc crust and the Pacific Plate. These rocks include partially to fully metamorphosed forearc basalts (FAB), island arc tholeiitic (IAT) basalts, mid-ocean ridge basalt (MORB) and ocean island basalt (OIB) [47–52].

Serpentinite mudflow matrix material from a wash core taken on ODP Leg 195 at Hole 1200B was wet-sieved for the 60-µm mesh fraction and had a far greater variety of metabasites (metamorphosed basalts) than were recovered in cores from ODP Leg 125 sites on Conical Seamount [43]. These amounted to a slightly greater total volume (approx. 12%) than those from Conical Seamount (approx. 10%) of this size fraction. Similar metabasites from dredged and cored (gravity and push cores from ROVs and submersibles) are consistent with low- to high-pressure, and low- to moderate-temperature origins [5,50].

The peak pressure and temperature conditions, from metamorphic paragenesis studies of metabasites from the Mariana forearc [5,50,51], are consistent with a plate interface source that is up to 18–19 km deep (approx. 0.6 GPa) and reaches temperatures of greater than 300°C [26,51] (table 1). These small fragments include crossite/white-mica/chlorite schist, chlorite schist, phengite schist, amphibole schist with zoned crystals the sodic amphiboles (including lawsonite, glaucophane and magnesioriebeckite); sodic-calcic amphiboles (dominantly winchite with less common barroisite); and calcic amphiboles (dominantly tremolite and magnesiohornblende), lawsonite schist, glaucophane schist and possibly jadeite schist. Minor mineral phases include sphene, epidote, hydrogrossular and albite [43,51,52].

## 6. IODP expedition 366 metabasites and reef limestones

### (a) Metabasite compositions

**Figure 4.**
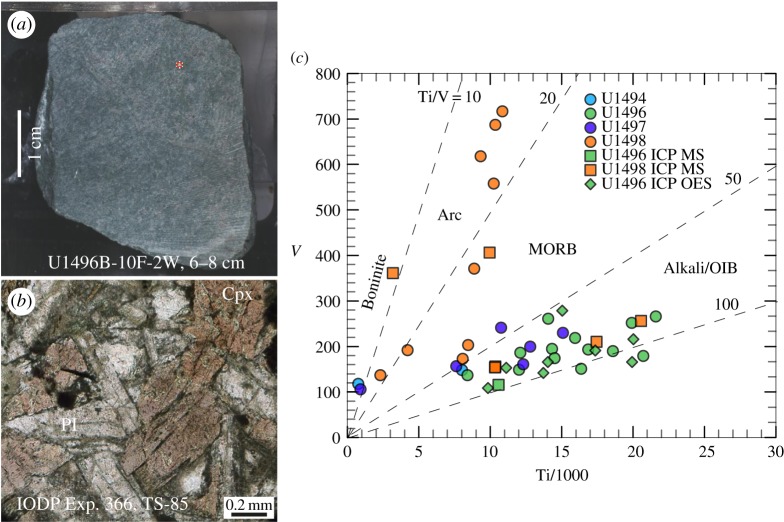
(*a*) Core section of metabasite (U1496B-10F-2 W, 6–8 cm). (*b*) Photomicrograph in plane-polarized light of metabasite in (*a*) with euhedral-subhedral titanaugite (Cpx) and plagioclase (Pl) with equigranular texture. Labelled, altered plagioclase shows relict albite twin. (*c*) Provenance plot of vanadium versus titanium/1000 [54] for metamorphosed basalts recovered from the IODP Exp. 366 Sites labelled in legend on figure. The points shown by circles were analysed by pXRF (shipboard, hand-held XRF), others were analysed as labelled (tables 2 and 3). Arc, Island arc tholeiitic basalt; MORB; mid-ocean ridge basalt; Alkalic/OIB,  ocean island basalt.

**Figure 5.**
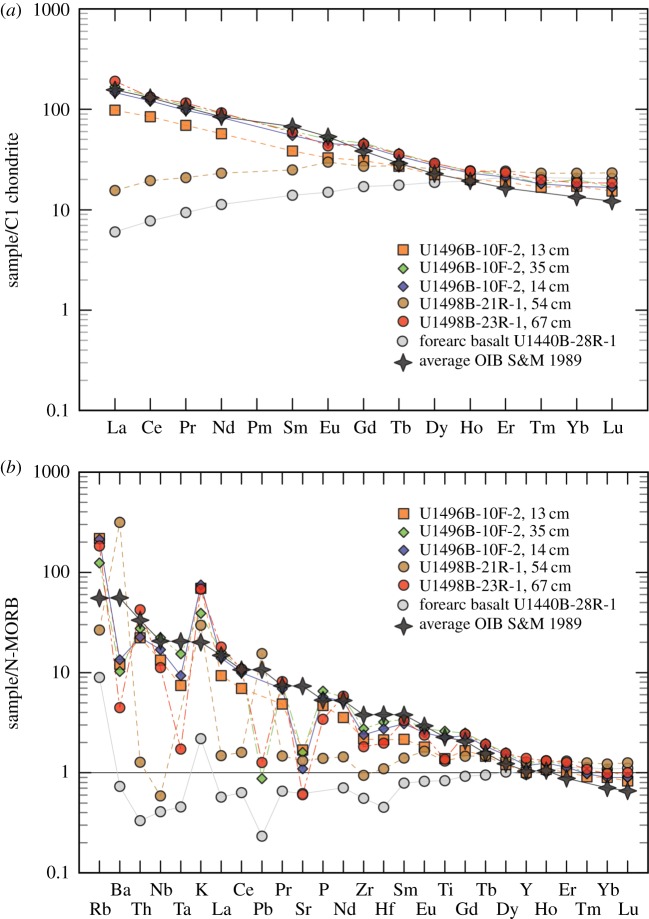
Plots of shore-based whole-rock Inductively coupled plasma mass spectrometry (ICP MS) analyses of trace element abundances. (*a*) C1 chondrite normalized rare earth elements; four of five samples are LREE-enriched, one is LREE-depleted. (*b*) Samples normalized to N-MORB for five metabasites, all (except U1498-21R-1, 54 cm, a MORB) show OIB compositional trends. Shown for comparison in both plots are a representative forearc basalt [55] and an average ocean island basalt (OIB) [52]. Normalizing values from Sun and McDonough (1989) [55]. For data, see table 2.

**Figure 6.**
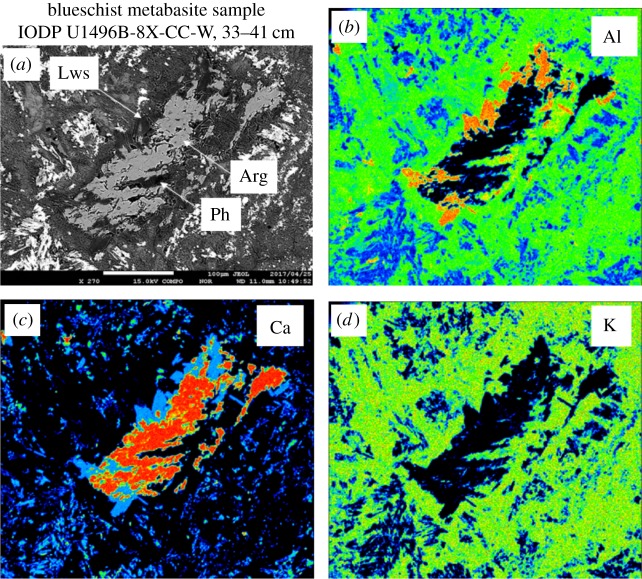
Imagery of microprobe element maps of the alkalic metabasite sample IODP U1496B-8X-CC-W, 33 to 41 cm, showing blueschist facies paragenesis with aragonite occurring as an interlocking mineral with lawsonite and phengite replacing a plagioclase crystal (elongate rectangle in centre of image. (*a*) Backscatter image of the polished, carbon-coated thin section (Lws, lawsonite; Arg, aragonite and Ph, phengite). (*b*) Aluminium elemental map of the same region as (*a*) showing Al distribution at margins of the aragonite crystal in orange. (*c*) Calcium elemental map of the same region as (*a*) showing Ca distribution in the aragonite crystal in orange. (*d*) Potassium elemental map of the same region as (*a*) showing K distribution ubiquitously distributed in former groundmass (green and blue phases surrounding the aragonite crystal are phengite and Na pyroxene, respectively) of alkalic ocean island basalt as tiny, scattered points of orange. (Analyst: Y. Ichiyama). (Online version in colour.)

Analytical methods used on the analysis of the metabasite rocks from all three seamounts drilled on IODP Expedition 366 (12/2016 to 02/2017) [3,53] are given in the electronic supplementary material associated with this paper. The IODP Expedition 366 Sites are located closer to the trench than the ODP Sites on Conical and South Chamorro Seamounts. All of the drill sites recovered metamorphosed basalt, with on-board analyses indicating mid-ocean ridge basalt (MORB) and/or island arc tholeiite (IAT) provenance [3,53]. IODP Expedition 366 lavas from Asùt Tesoru and Fantangisña Seamounts (figure 1) have V/Ti ratios in the OIB lava field (figure 4 and table 2). The depletion of V relative to Ti is a function of the fugacity (*f*o_2_) of a magma and its source, the degree of partial melting, and subsequent fractional crystallization [54]. Most IODP Expedition 366 metabasites are enriched in light rare earth elements relative to the heavy rare earths, but one is light-rare-earth depleted, similar to MORB or forearc basalt (figure 5*a* and table 3 [55]). MORB-normalized multi-element plots show that most samples have OIB-like patterns (figure 5*b* and table 3), with one similar to MORB (U1498B 21R-1) (compare with [56]). The negative Sr anomalies in the OIB-like samples indicate plagioclase fractionation, whereas the enrichments in fluid-mobile elements (K, Rb) indicate low-temperature alteration. These characteristics point to magma genesis from a locally enriched mantle source such as is abundant in Pacific Plate seamount provinces, but absent in the Mariana forearc [57,58]. The paragenesis of metamorphosed rocks of Pacific Plate origin collected from IODP Expedition 366 cores spans the gamut of metamorphic facies from zeolite [43], to greenschist [59], to blueschist [40,50,60] (figure 6).

**Table 2.** Ti and V analytical values used in figure 4. Offset = distance in centimetre from top of the core section (where not given, indicates numerous pebbles scattered in the section). pXRF = shipboard portable XRF. ICP-MS = ICP MS at Utah State University. ICP-OES = shipboard inductively coupled plasma optical emission spectrometry (ICP-OES) (for methods see electronic supplementary material).

method	samples analysed	offset cm	TiO_2_ wt%	Ti ppm	V ppm
pXRF	366-U1496B-8X-CC-W	2	2.39	14 334	195
pXRF	366-U1496B-8X-CC-W	7	2.00	12 010	149
pXRF	366-U1496B-8X-CC-W	0	2.42	14 509	174
pXRF	366-U1496B-10F-2-W	3	2.73	16 367	151
pXRF	366-U1496B-10F-2-W	7	1.40	8397	137
pXRF	366-U1496B-10F-2-W	27	2.66	15 934	219
pXRF	366-U1496B-10F-2-W	30	2.02	12 115	186
pXRF	366-U1496B-10F-2-W	36	3.45	20 712	179
pXRF	366-U1496B-10F-2-W	0	3.10	18 599	190
pXRF	366-U1496C-11R-1-W	30	2.81	16 867	193
pXRF	366-U1496A-3F-5-W	95	2.35	14 067	261
pXRF	366-u1496a-3f-CC-W	6	3.32	19 922	252
pXRF	366-U1496A-3F-5-W	99	3.60	21 587	266
pXRF	366-U1497A-7X-CC-W	28	0.15	905	106
pXRF	366-U1497A-11G-CC-W	10	2.52	15 095	230
pXRF	366-U1497A-11G-CC-W	10	2.05	12 309	161
pXRF	366-U1497A-12F-1-W	78	2.14	12 807	200
pXRF	366-U1497A-12F-1-W	91	1.80	10 765	241
pXRF	366-U1497A-13G-CC-W	4	1.27	7626	157
pXRF	366-U1498B-8R-1-W	12	0.38	2291	137
pXRF	366-U1498B-21R-1-W	38	0.70	4208	192
pXRF	366-U1498B-21R-1-W	52	1.56	9331	618
pXRF	366-U1498B-21R-1-W	63	1.73	10 346	687
pXRF	366-U1498B-21R-1-W	75	1.48	8886	371
pXRF	366-U1498B-21R-1-W	103	1.71	10 244	558
pXRF	366-U1498B-21R-2-W	71	1.41	8452	203
pXRF	366-U1498B-23R-1-A	39	1.35	8078	173
pXRF	366-U1498B-21R-1-W	54	1.81	10 841	717
pXRF	366-U1494A-3F-2-W	61	1.34	8025	148
pXRF	366-U1494A-3F-2-W	81	0.12	749	118
ICP-MS	366-U1496B-10F-2, 13 cm	13	1.77	10 589	116
ICP-MS	366-U1498B-21R-1, 54 cm	54	1.66	9947	406
ICP-MS	366-U1498A-3R-2, 80 cm	80	0.53	3178	361
ICP-MS	366-U1496B-10F-2, 35 cm	35	3.43	20 573	256
ICP-MS	366-U1496B-10F-2, 14 cm	14	2.91	17 435	210
ICP-MS	366-U1498B-23R-1, 67 cm	67	1.73	10 357	156
ICP-MS	366-U1498B-23R-1, 67 cm^a^	67	1.72	10 332	154
ICP-OES	366-U1496B-10F-2, 35-38 cm	35	3.34	20 025	216
ICP-OES	366-U1496B-10F-2, 17-20 cm	17	2.9	17357	191
ICP-OES	366-U1496B-10F-2, 5-8 cm	5	1.85	11 113	153
ICP-OES	366-U1496B-10F-2, 13-21 cm	13	1.64	9845	109
ICP-OES	366-U1496B-10F-2, 0-5 cm	0	3.33	19 942	166
ICP-OES	366-U1496B-8X-CC, 0-4 cm	0	2.51	15 056	279
ICP-OES	366-U1496B-8X-CC, 8-13 cm	8	2.34	14 030	166
ICP-OES	366-U1496B-8X-CC, 34-31 cm	34	2.29	13 711	142

^a^duplicate

**Table 3.** Trace element concentrations in ppm for samples shown in figures 4 and 5. Inductively coupled plasma mass spectrometry (ICP-MS) analysis at Utah State University, for methods see electronic supplementary material (analyst, Andrew Lonero).

element	standard BIR-1	standard BHVO-1	IODP-366- U1496B-10F-2, 13 cm	IODP-366- U1498B-21R-1, 54 cm	IODP-366- U1496B- 10F-2, 35 cm	IODP-366- U1496B- 10F-2, 14 cm	IODP-366- U1498B- 23R-1, 67 cm	IODP-366- U1498B- 23R-1, 67 cm^a^
Ti	6941	17 036	10 589	9947	20 573	17 435	10 357	10 332
V	424.5	356.3	115.7	406.3	256.5	209.9	156.0	153.6
Rb	—	7.4	118.5	11.5	66.1	115.9	99.5	100.0
Sr	130.1	408.5	153.0	118.3	145.1	96.9	52.2	53.0
Y	15.1	23.1	25.3	33.0	31.5	30.3	36.0	36.5
Zr	16.9	177.6	226.8	96.3	280.4	253.7	191.2	191.7
Nb	—	12.7	24.1	—	45.0	32.5	19.1	19.1
Cs	—	0.3	3.7	0.3	1.4	4.1	2.4	2.4
Ba	5.8	137.3	69.2	2007.9	58.2	77.7	21.5	21.4
La	0.3	16.3	20.2	0.6	36.7	31.9	42.0	42.7
Ce	0.2	37.9	48.9	8.9	80.3	71.5	78.2	80.0
Pr	0.6	5.8	6.4	1.9	10.1	9.1	10.7	10.9
Nd	3.1	25.9	26.1	10.6	41.6	37.8	42.3	43.0
Sm	1.4	6.6	5.7	3.7	9.0	8.1	8.7	8.7
Eu	0.7	2.3	1.8	1.7	2.8	2.6	2.4	2.4
Gd	2.3	6.8	6.1	5.4	9.2	8.7	9.0	9.3
Tb	0.5	1.2	1.0	1.0	1.3	1.2	1.3	1.3
Dy	3.0	5.6	5.5	6.3	7.2	6.8	7.1	7.3
Ho	0.7	1.2	1.1	1.3	1.3	1.3	1.3	1.4
Er	2.1	2.8	3.0	3.9	3.5	3.4	3.8	3.7
Tm	0.3	0.5	0.4	0.6	0.5	0.4	0.5	0.5
Yb	1.9	2.1	2.7	3.7	3.2	2.7	3.0	3.0
Lu	0.3	0.5	0.4	0.6	0.4	0.4	0.5	0.5
Hf	0.5	4.4	4.4	2.2	6.6	5.6	4.1	4.2
Ta	1.1	1.3	1.0	—	2.0	1.2	0.2	0.4
W	1.3	0.8	—	—	0.5	0.4	—	—
Pb	—	1.7	—	4.6	0.2	—	0.6	0.5
Th	0.1	1.4	2.7	0.2	3.3	2.7	5.1	5.2
U	—	0.6	0.6	—	1.3	0.8	0.8	0.9

^a^Repeated analysis.

### (b) Limestone compositions

**Figure 7.**
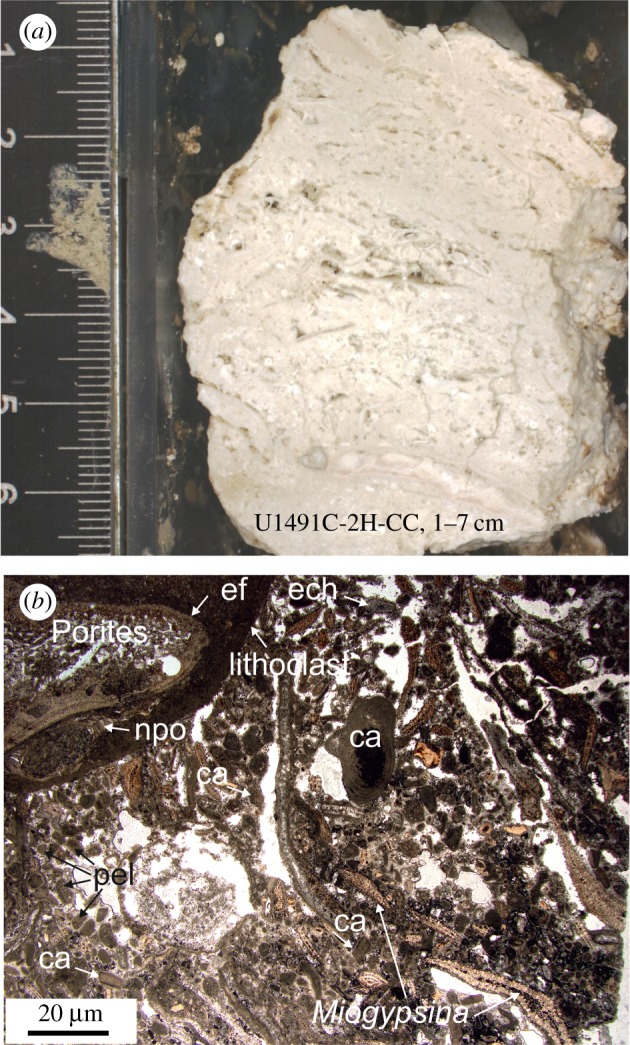
(*a*) Miogypsina rudstone cobble with larger lithoclasts and coralline, red-algal grainstone) matrix from Yinazao Seamount flank drill core. (*b*) Photomicrograph (plane-polarized light) of U1491C-2H-CC, 1–7 cm at 25 mbsf (see text for description). ca, coralline red algae; ech, echinoderms; pel, Peloide (all micritic round clasts); ef, encrusting foraminiferan; npo, neopycnodontid oyster). (Analyst: W. Kurz). (Online version in colour.)

Limestone breccia and cherty-limestone fragments were also recovered from the IODP Expedition 366 cores [53]. Cores from Yinazao Seamount recovered the first pieces of reef material (figure 7) from a subducted Pacific Plate seamount from one of the forearc serpentinite mud volcanoes. The reefal cobbles recovered from Core U1491C-2H-CC-1–7 cm are, in fact, the first pieces of reef material ever collected from a subducted plate seamount in the world (figures 1 and 5*a*,*b*). The cobble analysed in detail is a Miogypsina rudstone with larger lithoclasts and coralline, red-algal grainstone matrix. The matrix includes echinoderm, bryozoan and decapod fragments, small benthic and a few planktonic foraminifera. The lithoclasts consist of packstone with large bioclasts such as a Halimeda plate or a Porites fragment. The latter is encrusted by a sessile foraminifer and a neopycnodont oyster. Although an alkalic basalt dredged from Quesada Seamount immediately outboard of the trench axis, at the latitude of Yinazao Seamount, was dated at 129.3 + 2.6 Ma [61], no reef material has been collected there with which to compare ages with subducted seamount rocks collected on IODP Expedition 366. Although the proximity of our mafic OIB samples from Yinazao Seamount suggest a Cretaceous origin for the samples, we do not yet have age dates from any of the mafic samples with which to verify this. The presence of Miogypsina suggests a much younger age, early to middle Miocene, for the reef limestone recovered. Clearly, this assemblage represents a shallow water (photic-zone) environment. Our interpretation is that it is most likely derived from the uppermost part of a Cretaceous Pacific Plate seamount's reef sequence.

## 7. Discussion

### (a) Significance of intact subducting seamount

The observation of a large expanse of intact, reefal capping sequences on the inner Mariana Trench slope, during Dive 4 of the R/V Okeanos Explorer's 2016 expedition ‘Deepwater Exploration of the Marianas’, implies that Cretaceous Pacific Plate seamounts can be subducted without significant rotation or shearing. The Mariana convergent margin has no large accumulation of sediment, such as that which forms at accretionary convergent margins. The forearc mantle is exposed below depths of about 6 km along the extent of the Mariana convergent plate margin where it has been observed and/or sampled. An impinging seamount must, therefore, contact forearc upper mantle and crustal sequences immediately as it enters the Mariana subduction channel. Because the reef sequences observed on ROV Dive 4 show no deformation, we infer that deformation must be restricted to the contact edges of such edifices with the adjacent forearc rock or to deeper regions of the subduction channel. We also infer that the morphology of the forearc near the position of Dive 4 is a result of uplift resulting from subduction of the seamount.

It is possible that the eastern half of the Mariana forearc mantle has been serpentinized over broad areas, lacks cohesion and has a low coefficient of friction when permeated by fluids at depth [62,63]. It may be faulted sufficiently so as to deform readily during incursion of a subducting edifice. Thus, the contact zone between subducted plate and forearc wedge must be lithologically complex and may have significant relief.

The forearc west of the location of ROV Dive 4 is up to 4 km shallower than the average depth along strike. There is no multi-channel seismic (MCS) reflection data in this region, but MCS profiles across other areas of near-trench forearc uplift have failed to show evidence of underlying subducted seamounts [64], even where IODP Expedition 366 drill cores recovered reefal limestone clasts in serpentinite mudflows.

### (b) Exhumed reef fragments

Assuming a Pacific Plate convergence rate of approximately 3.5 cm per year [65,66] for the southern Mariana forearc region, the hypothetical position of the seamount from which the reef cobble (in Core U1491C) derived was 400 to 700 km east–southeast of the trench in early to middle Miocene times when the equatorial Pacific carbonate compensation depth was lower than today [67]. Most likely its position was outside the outer-trench-rise. Using the current Pacific Plate convergence rate, and back-tracing approximately 550 km, the seamount would have been located in the area of today's Micronesia atolls where appropriate shallow water conditions still occur. Because the cobble from Core U1491C was recovered at a depth of over 4500 m below sea level, and from within a serpentinite mudflow, it must have been brought up from greater depth in the subduction channel. The depth to the subduction channel from MCS data at this location is 14 km below sea level [64]. The largest of the seamounts and guyots east of the Mariana Trench are approximately 4–5 km taller than the surrounding sea floor. Assuming that the seamount was subducted with minimal reshaping, the reefal cobble could have been derived from approximately 9–10 km beneath the forearc sea floor before being entrained in rising serpentinite mud. Temperatures and pressures at such a depth would be less than 80°C and approximately 0.3 GPa (table 1).

The exhumation of seamount lavas and unaltered reefal fragments containing shallow-water fauna thus indicates that materials from shallower in the subduction channel than the presumed abyssal depths of the subducting Pacific sea floor can be recycled through the forearc. Clearly, such materials would have experienced lower pressures and temperatures than the abyssal seafloor during subduction and would likely be below the sterilization temperature and depth conditions for microbial communities extant within these materials.

**Figure 8.**
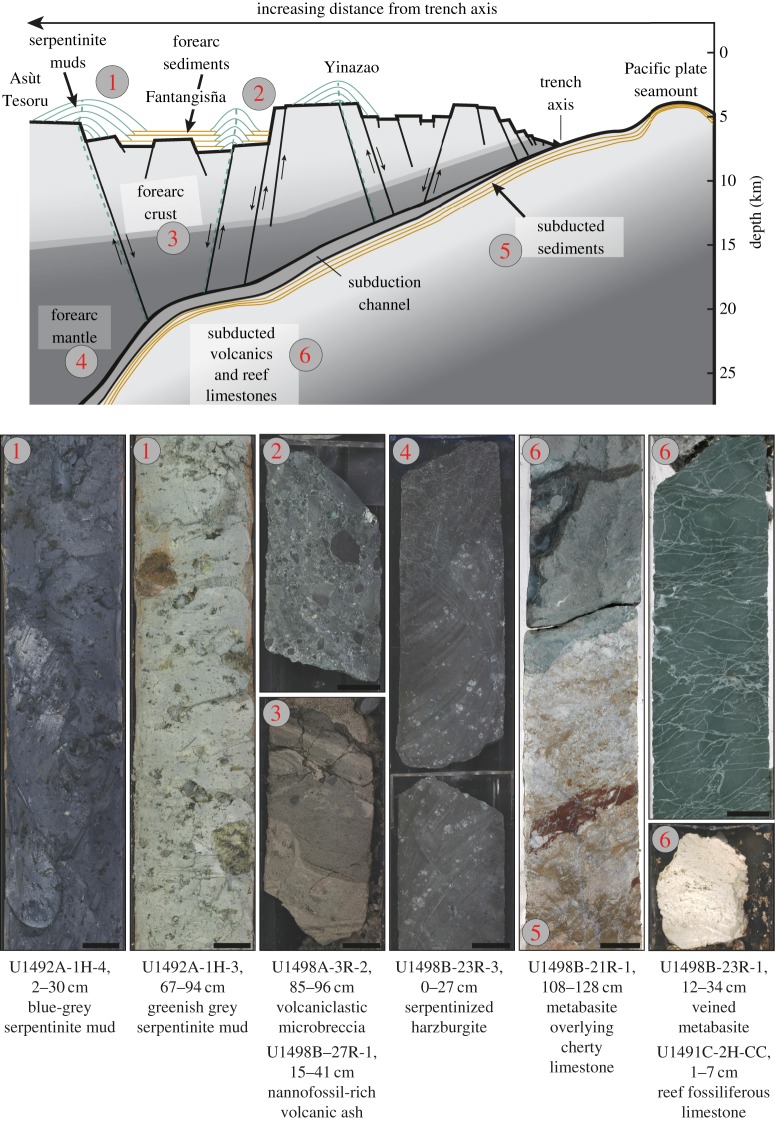
Idealized west–east cross-section of various Mariana forearc settings, including the relative positioning of serpentinite mud volcanoes and representative cored materials from each setting [3] (top figure modified after Fryer *et al*. [5]). (Online version in colour.)

The discovery of an intact expanse of a Cretaceous reefal section in the deep inner slope of the Mariana Trench makes incorporation of subducted seamounts into nonaccretionary forearc regions indisputable. The inclusion of pieces of oceanic plate and plate seamounts in serpentinite mudflows (figure 8) in the Mariana forearc indicates that rocks can be derived at any depth, from the contact with abyssal sea floor, from fault zones that border contact between subducted seamounts and adjacent forearc faults, or from regions where dismembered seamount fragments have been left behind as the main edifice is subducted.

### (c) Forearc deformation and eruption triggers

Although we have now seen that seamounts can be accommodated without deformation into non-accretionary convergent margins, controversy exists regarding whether subducting seamounts prevent earthquakes, facilitate them [29,31,68–70] or cause erosion of the base of the overriding plate. Results of several modelling studies [71–73] and high-resolution bathymetric data [74], indicating forearc deformation linked with subduction of seamounts and ridges, have been argued both ways. If we examine the distribution of seismicity from 1900 until 2017 at depths less than 50 km in the Mariana forearc (figure 1), we see that seismic events are located near some serpentinite mud volcanoes, but not near others. Thus, the currently known spatial association of these events with known serpentinite mud volcanoes is not sufficient to identify a temporal relationship between serpentinite mud volcano activity and likely processes of seamount subduction. A temporal relationship between large clusters of earthquakes within the subduction channel and shallow events near a mud volcano has, at least in one locality, been demonstrated [4], however.

The spatial relationship between forearc fault scarps or lineaments and the distribution of serpentinite mud volcanoes is well known [5,32,35,37,41,42,51,75]. At shallow levels in the forearc crust, multi-channel seismic data [76] show considerable faulting within forearc sediments in the 100 km proximal to the trench. A greater degree of faulting at millimetre to metre scales was observed during Deep Sea Drilling Project Leg 60 in cores at Sites 458 and 459 on the eastern Mariana forearc at approximately 18°N [77]. More recent multi-channel seismic data [75] provides evidence of faulting within the upper crustal sequences of the Mariana forearc, but there are no data that define faults within the forearc mantle wedge.

An important approach to assessing the stress state of the subduction channel, on a fine scale, is by measuring the over-pressuring of fluids in the subduction channel as a forcing function to stimulate seismic activity. Within the Mariana forearc, over-pressure is transmitted in fluids released from the subducting plate initially through pore collapse and then by dehydration of hydrous minerals. Numerous studies have suggested a relation between seamount subduction and over pressuring of forearc fluids [28,29,31,68,70]. A borehole observatory emplaced in ODP Hole 1200C recorded pressure fluctuations that were triggered by two large (Mw 7+) earthquakes during a two-year monitoring period [78].

Including ODP Hole 1200C on South Chamorro Seamount, there is now a transect of borehole observatories on summits of four serpentinite mud volcanoes, each penetrating an active hydrologic zone. Once the IODP Expedition 366 cased boreholes are fitted with CORK-Lites [79] it will be possible to instrument the seamounts to monitor a range of physical and hydrologic properties, correlate them with regional seismicity, and conduct manipulative experiments to assess active processes within the conduits of the mud volcanoes and the underlying subduction channel. Studies of the crystal preferred orientation of relict minerals in serpentinized peridotites of the Mariana convergent plate margin permits the interpretation of the stress history of the suprasubduction-zone mantle wedge [80].

Rock clasts entrained in serpentinite mudflows provide the best evidence to evaluate the regional state of stress of the upper Pacific Plate in the Mariana subduction channel and the overlying forearc lithosphere. At a contact between a metabasite and a cherty limestone (figure 8), in core U1498B-21R-1 through U1498B-21R-2, we observed cataclastic faults and extensional structures (high-angle normal faults and extensional veins) (figure 8). These may be related to: (1) bending of the lower plate during subduction; (2) deformation within the subduction channel; and (3) accretion and vertical tectonism of pieces of the subducted plate and seamounts into the forearc region. Where seamounts are subducted, the likely heterogeneity of structural and stress environments may limit the dimensions of fracture propagation [29]. Stress events are likely frequent, leading to seismic creep within the zone of contact between subducting seamounts and the surrounding forearc.

### (d) Morphology of the subduction channel

The admixture of fragments of shallow reef limestones, alkalic OIB lava fragments, and forearc lavas within serpentinite mudflow drill cores permits us to speculate that the contact between the subducting edifices and the surrounding forearc has higher relief than previously conceived. Although the maximum pressure–temperature ranges of the metabasites can be estimated based on distances from the trench axis, the broad range of metamorphic conditions of rock clasts from within each mud volcano indicates that metabasites can be plucked from considerable depth ranges along the conduit walls, including the abyssal depths of the subducted plate or the flanks of a seamount edifice subducting with it. In contrast, dredges that were collected on a scarp closer to the trench, near Conical Seamount, recovered low-pressure/low-temperature (greenschist-facies) metamorphosed basalts of these same provenances, as well as chert and limestones [47,81]. If the leading edge of the overriding plate is weak, the contact zone between the impinging edifice and the forearc will be the preferred zone of weakness for the development of a mud volcano conduit. Conduit development would be most likely to occur at the contact between a relatively strong edifice structure and a weak surrounding serpentinized forearc mantle wedge (figure 8).

### (e) Implications for microbial exhumation

If the depth traversed during the exhumation of a mudflow unit in a given eruptive episode varies greatly, a conduit at the edge of a subducted seamount could tap into a range of pressure and temperature conditions at the boundary between forearc mantle and a subducting edifice. The largest of the Pacific Plate seamounts east of the trench are flat-topped guyots, as much as 100 km in diameter and, as the reefal material recovered indicates, were close to sea level in the Miocene. So, they would have been close to 4–5 km high. The region of the forearc wedge, on which the serpentinite mud volcanoes rest, is from approximately 13–19 km thick (table 1). A large subducting seamount could perturb the thermal structure of the outer forearc wedge into which it would have been subducted. We suggest that although the adjacent, deep subducting basaltic crust may experience temperatures in excess of the limits for life, shallower, cooler segments of the down-going plate, such as the flanks and summit of a subducted seamount, could still harbour viable microbial communities. Such communities, when they contact fluids associated with serpentinizing forearc mantle, would likely shift toward those in favour of organisms best suited to the high pH and reducing conditions in these fluids (e.g. [82–85]), and thus become dominant members of the community. These then would persist near the active summits of the serpentinite mud volcanoes today. This scenario is consistent with observations of the type of microbial communities observed thus far at the summit springs on several Mariana serpentinite mud volcanoes [13,15].

The results of IODP Expedition 366 show that the distribution of serpentinite mud volcanoes that erupt fragments of Pacific Plate and its seamounts extends over 600 km along-strike and over 90 km across-strike of the southern Mariana forearc. There are also serpentinite seamounts atop a ridge 50 km wide proximal to the Izu-Bonin Trench that extends for the entire length of that convergent margin, though they are apparently not currently active [42].

### (f) Implications for the early earth environment

Although the Mariana serpentinite mud volcanoes are the only known currently active sites of serpentinite mud eruptions, deposits of serpentinite materials of similar type are observed world wide in convergent margin deposits with ages as old as Early Paleozoic (approx. 480 Ma) [4,5,86,87]. The occurrence of serpentinite deposits in the Isua formation of southwestern Greenland that are similar to the Mariana forearc serpentinite mudflows [88], are of particular note, and they are of Eoarchean (3.81 to 3.70 Ga) age. Thus, the phenomenon of convergent margin serpentinite mud volcanism has an apparently long and spatially broad history in the Earth system.

Ever since hydrothermal vents on mid-ocean ridge systems, teeming with life, were discovered, the focus on a chemosynthetic, hydrothermal origin for life on Earth has concentrated on the oceans. Over the past few decades, research into the environmental conditions conducive for life's origin has increasingly suggested these could have occurred in close association with serpentinization [89–103]. Many of these researchers have studied environments near mid-ocean ridges. Bada [104] has suggested this would be a problem if hyperthermophilic organisms do not form the root of the tree of life. Furthermore, it is established [105,106] that the high temperatures of hydrothermal systems would have precluded sufficient temporal stability of prebiotic macromolecules necessary for the development of living cells on the Hadean/Archean Earth. Most responses to this argument have been that, although at high temperature vents, conditions for the evolution of life would have been unlikely, only millimetres away the temperatures would have been cool enough to permit it. Even so, it is possible that the inherently episodic and ephemeral nature of hydrothermal systems [107] imposed a temporal constraint on the likelihood of life's beginning at Earth's hydrothermal systems. Furthermore, longevity and dispersal of the earliest life forms from hydrothermal vent environments would have been impinged upon by proximal hydrographic and regional geologic impediments, as they are today [108]. Some researchers [109–112] prefer models in which subaerial, shallow hydrothermal systems with fresh-water and drying/hydrating cycles would help to increasingly concentrate potential reactants. These too may suffer the difficulties with stability of progenitor molecules as in other hydrothermal systems. Relatively cooler, but temporally and spatially more stable environments in the early history of the Earth, where serpentinization was prevalent, would be a likely alternative. Ancient subduction zones are an obvious possibility, if some form of plate tectonics existed in antiquity.

Arguments continue regarding when plate tectonics first began on the Earth (e.g. see reviews [113,114]), and thus when plate margins (subduction zones and mid-ocean ridges) and associated volcanism first formed. Zones of lithospheric plate convergence, represented today by some ancient (Archean: 4–2.5 Ga) greenstone belts exist throughout the continents. Furthermore, the results of *δ*^18^O analysis of zircon crystals, with ages of up to 4.4 Ga [115,116], indicate that surface temperatures were cool enough in the late Hadean (4.4–3.82 Gya) for liquid water to have existed on Earth's surface and thus for life to have evolved [117]. Arguments regarding the possibility of initiation of plate tectonics in the Hadean (greater than 4 Ga) require, at minimum, surface water underlain by a dry mantle [113]. If subduction processes were occurring on the early Earth, serpentinization in suprasubduction channels would have been possible.

Today as we observe mid-ocean ridges, we see their segmentation varying on scales from tens to hundreds of kilometres [118,119]. Today's trenches stretch for thousands of kilometres (e.g. Mariana–Izu–Bonin, Peru–Chile and Aleutian Trenches) and have persisted for tens to hundreds of millions of years. They are the more temporally stable plate tectonic features, are cooler regimes and can provide a widespread contiguous serpentinization environment.

### (g) Subduction zones and the origin of life

The Isua Formation of southwestern Greenland is garnering increasing interest as a suggested Eoarchean intraoceanic convergent margin [120,121] setting. The discovery of serpentinite deposits that are interpreted to indicate serpentinite mud volcanism in an Isua Formation forearc setting [88] pushes the formation of such mud volcanoes back to near the Late-Hadean/Early-Archean boundary. The chemical and physical characteristics of today's serpentinite mud volcanism in the Mariana forearc conform to many of the suggested requirements for optimal conditions of the development of life. The fluids emanating from these mud volcanoes are freshened relative to seawater [24]. The eruptive pattern is episodic at each edifice, and they persist over time scales of tens of millions of years [4,5,35]. The muds contained in the upper, active portions of the conduits are bathed in vent fluids during each eruptive cycle, but may undergo periods when there is no fluid flux. These dormant periods can concentrate precipitates within the porous mudflows. The conduit regions currently support active microbial communities [14,15]. As we have shown here, the conduits of all sampled thus far contain a broad range of clast lithologies. The rising muds, therefore, have the potential to contain a wide variety of reactants. Thus, if such mud volcanism can be considered a suitable serpentinite environment for the beginnings of chemosynthetic life on Earth, how early could this process have occurred?

Oxygen isotopic compositions of Hadean (4.4 Ga) zircons [115–117] imply that temperatures of the early Earth's surface were such that oceans could have existed. Data support fluid-flux melting of the mantle at least by Eoarchean (3.7 Ga) time [121]. The early formation of continental crustal masses [113–121], and of putative off-ridge plate seamounts formed by localized pressure-release melting or hot-spot processes, and their collision at convergent plate margins argues for the potential subduction of high-relief features. If so, then serpentinization juxtaposed to fault bounded, protocontinental or seamount structures in ancient subduction-zone mantle could have been possible anywhere along thousands of kilometres of convergent plate margins at the Hadean/Archean boundary.

Subduction of high relief protocontinents or seamounts would have had the potential to form complex subduction channels, and to result in vertical tectonic deformation in forearc regions, deep penetrating faults, and contact between serpentinized mantle and compositionally complex features on the subducting plate. Thus, development of egress channels for deep-derived fluids, and the eruption of mudflows on the sea floor, composed of comminuted and remobilized serpentinite and fault gouge with fragments of subducted lithospheric lithologies, could have occurred. We agree that it is valid to consider serpentinization environments as ones likely to have promoted the transition from prebiotic conditions to the evolution of living cells. However, we suggest that envisioning such environments as limited to regions of hydrothermal systems on mid-ocean ridges, or in hot springs settings on land, is too narrow a view.

## 8. Conclusion

Our observation of an intact section of Cretaceous reef exposed in the Mariana Trench inner slope shows, for the first time, that large expanses of impinging seamounts can remain undeformed at first contact. It also confirms uplift of the surrounding forearc region. We observe that forearc relief is enhanced where thicker subducting crust or large seamounts impinge, thus adding to the vertical tectonic deformation of the forearc region. ODP and IODP drill cores from five serpentinite mud volcanoes contain a range of subducted plate and seamount lithologies. The mudflow matrix composition and the broad variation in degree of metamorphism of the rocks recovered suggest that the relief of the subduction channel beneath the Mariana forearc is likely much higher than generally thought.

High relief in the subduction channel would affect the temperature and pressure conditions, as well as mineralogical and pore fluid compositions of muds that erupt at the active Mariana forearc serpentinite mud volcanoes. A given eruption may involve materials plucked from conduit walls at a variety of depths. This can explain the wide range of degree of serpentinization of ultramafic clasts contained in the mudflows and the wide range of metamorphic facies and degree of deformation observed in metabasites derived from the down-going Pacific Plate and subducted seamounts. If subducting seamounts pass through the shallow to intermediate depths of the subduction channel relatively intact, microbial communities present in the sub-seafloor on their flanks or within the abyssal Pacific Plate's lithospheric slab could also be exhumed from varying depths. Serpentinization of forearc mantle fault gouge, in contact with rising fragments of the subducted plate, likely selects for those microbes that prefer the resulting high pH (up to 12.5) and reducing conditions of the serpentinizing pore fluids [13].

Researchers have suggested a variety of serpentinization environments that may be supportive of the earliest formation of life [82–85,89–99]. These mainly stress hydrothermal vent environments. The focus on mid-ocean ridges and off-ridge hydrothermal regions results from discovery of vast accumulations of vent fauna in the oceans today. The focus on subaerial hydrothermal pools near volcanoes stresses the hydration/dehydration potential for concentrating prebiotic molecules. However, hydrothermal activity is sporadic and ephemeral on geologic time scales along mid-ocean ridges, and is spatially miniscule by comparison with the 70% global expanse of the ocean floor. The environment of shallow (approx. 0–20 km) subduction at convergent plate margins is cooler, more contiguously widespread and supports optimum geochemical conditions over geologically greater timescales. So, perhaps we must ask what other geologic settings would have been conducive to enabling the transition from the milieu of probiotic molecules to the development of living cells. If we consider the possibility of the presence of an ocean on the early (4.5 Ga) Earth and the advent of cyclic crustal generation and consumption via some early form of plate tectonic activity, then the temporal continuity and spatial breadth of convergent plate margins is likely far greater than that of hydrothermal vent locations. As we ponder the most favourable environments for the development of chemosynthetic life on the basis of serpentinization, either on Earth or other planets, it would be well to consider processes other than those just of hydrothermal systems.

Even discarding speculations about ancient environments, the causes of and conditions during current, active episodes of escape/venting of material from the subduction channel in any phase of plate subduction remain uncertain. If seismic events trigger episodes of eruption at modern serpentinite mud volcanoes, is it possible to take the pulse of subduction? Cased boreholes left at the summits of four of the drilled ODP and IODP Sites at summits of four serpentinite mud volcanoes provide a regional laboratory for the study of geophysical, geochemical and geobiological processes associated with the subduction channel beneath the Mariana forearc. The planned and funded deployment of instrumentation in these seafloor observatory holes will permit further analysis of interrelationships between seismic activity, pore fluid flux, eruption mechanics and effects on microbial communities of the serpentinite mud volcanoes. The results will impact current paradigms of lithospheric deformation, mass cycling and physical conditions at the plate contact zone and help define potential constraints on the mechanism by which subsurface microbes could be exhumed from the subduction channel and ‘recycled’ without being subjected to sterilizing conditions.

## Supplementary Material

Supplementary Materials

## Supplementary Material

Table S1

## Data accessibility

The analytical methods text and an additional table with data supporting the major and trace element data in this article have been uploaded as part of the electronic supplementary material.
