# Supplementary material for: Mariana serpentinite mud volcanism exhumes subducted seamount materials: implications for the origin of life
Source: Philos Trans A Math Phys Eng Sci. 2020 Jan 6;378(2165):20180425. doi: 10.1098/rsta.2018.0425 (PMC7015305; doi:10.1098/rsta.2018.0425)
Supplement: Supplementary Materials [file rsta20180425supp1.docx]

**Electronic Supplementary Materials**

**Analytical Methods:**

Analytical methods for trace element analyses determined by ICP-MS at Utah State University Geochemistry Lab (shown in Tables 2 and 3 of the main text) are as follows:

Samples were digested with nitric and hydrofluoric acids and analyzed on a Thermo XSeriesII ICP-MS system using standard methods. All elements were determined in fully quantitative mode with external and internal standardization. Where multiple isotopes of elements were analyzed, results were averaged except in the case of zirconium where only 90Zr was reported. BIR-1 and BHVO-1 are basalt standards that were run with these samples through the entire procedure and agree well with accepted values. Sample 863-6191 was replicated as an indication of precision. Dash marks (-) indicate values measured were below those of the method blank. Average percent error on trace elements compared to accepted values for BIR-1 were about 0.5% of theoretical.

Analytical methods for elemental analysis by pXRF of rock samples at sea on the *JOIDES Resolution* (Table 2 in main text) are as follows:

A new shipboard portable XRF (pXRF), an Olympus DeltaX handheld instrument, was acquired by IODP for use on Expedition 366. The Olympus DeltaX is a self-contained energy-dispersive XRF survey instrument with data correction packages tailored to geological applications (geochemistry and soils). Data correction is based on the “fundamental parameters” methodology, solving a series of non-linear equations for each analyzed element that are based on metrics for the X-ray source, fluorescence intensities, absorption coefficients, absorption edge effects for each wavelength analyzed, parameters for sample geometry [1], and a Compton normalization scheme [2]. On IODP Expedition 366 we primarily employed the "geochemistry/soils" protocol that presumes a perpendicular sample geometry and analyzes for elements at several different filter settings to optimize results.

Samples removed from the core were placed in a specially made shielded sample analysis assembly. Analysis *in situ* was done with a shielded sleeve with analyzer mount. Powered samples of basalts were analyzed using standard inductively coupled plasma–atomic emission spectroscopy (ICP-AES) technique [3] to provide a measure of the concentrations of selected trace elements (Ni, Cr, Sr, Ba, Sc, V, and Zr) and the pXRF was used to determine concentrations of Fe, Ca, Mn, Ni, Cr, Zn, and Sr.

In the case of *in situ* samples, a layer of 355 Ultralene 0.16 mil (4μ) thin film was placed over the core to protect the face of the analyzer. The geometry of the sample was consistent, with its surface close and parallel to the face of the analyzer, thus minimizing atmospheric absorption effects and geometry-related losses.

- Samples were selected based on adequate size for analysis (approximately 4 cm2 or greater), minimum spacing within the core so as not to duplicate numerous measurements within the same interval, differences in observed physical properties (i.e., color, texture, grain size, etc.), suitable smooth edges (preferably cut), and proximity to zones of alteration, change in lithology, or other distinguishing features.

- Surfaces were dried with an absorptive material (KimTech Science brand wipes) to improve signal penetration depth.

- Sample identification, descriptions, etc., were recorded on the shipboard pXRF lab checklist for later use in describing the samples and/or reproducing the measurements.

The Delta Advanced PC software was initialized on the chemistry laboratory notebook PC (Dell XRF Host). A calibration check was performed before initial use, and approximately every ten hours or when prompted by the software. Samples were measured manually for 60 seconds, with three measurements per unknown constituting a single analysis. One powdered standard reference material (BHVO-2) was analyzed with each set of unknowns to track machine performance. The total variation among individual measurements of the same sample was always well within the measurement uncertainties reported by the instrument and was often ≤ 5%. Day-to-day variation in our results for reference material BHVO-2 indicated no more than ±3% variability for higher precision elements over the course of analytical work during this Expedition

A primary use of the pXRF instrument was to conduct quick geochemical assessments of cut rock surfaces in the cores and of thin section billets made onboard. It was also used to assess elemental abundances in powdered samples and fusion beads. The beads are made by fluxing sample powders with LiBO_2_. The XRF powder mount assemblies provided with the instrument wer used. A short length of Ultralene 4 micron film was used to seal the mount, and a plastic ring-cap to give a smooth, transparent surface for sample loading. A 2-3 mm layer of sample powder was loaded in the mount. A small circle of filter paper (Whatman 24 mm circles, grade 540) covered the sample, and either a round 24 mm plastic foam spacer or plastic floss was used as packing to hold the sample powder in place, followed by a snap-on plastic sealing cap. The transparent surface of the mount was placed face-up in the XRF sample holder. Fusion beads prepared for ICP-AES measurement were placed directly on the pXRF observing window in the shielded mount.

Calibration/correction curves for the different elements measured via pXRF were determined using the same suite of standard reference materials used for ICP-OES analyses. Slope and intercept values from calculated working curves were used to produce concentration results using Microsoft Excel software after analysis of the samples was completed. Slopes and intercepts were obtained by plotting the intensity of the signal versus the known concentration of the standards analyzed. The intensities measured by the pXRF were then used to determine concentrations of specific elements within the section of the rock analyzed. Rocks and powder samples were run against calibration standard powder mounts [4], and fusion beads were calibrated against a set of fused standards.

The elements measured via pXRF were Mg, Al, Si, S, Ca, Cr, Mn, Fe, Ni, Cu, Zn, and Sr. Data for elements with correlation curves showing substantial offsets in their [y] intercepts, and for elements with correlation curve r values of less than 0.95, were not utilized in our interpretations. For the remaining elements, the correlation curves became the basis for working curves to calculate accurate elemental concentrations. These curves are reliable over the concentration ranges constrained by our reference materials.

Analytical methods and parameters for major element microprobe analytical standards, operating conditions and error analysis for data presented in Supplementary Table 1 (see: Supp_Material_Table_1.xlsx) are as follows:

The standards were: Garnet, Verma (Mn) for Mn kα; Sphene glass for Ti kα; Chromite USNM 117075 for Fe kα, Mg kα, and Cr ka; Diopside-2 (UCLA) for Ca kα and Si kα; Albite, Amelia for Na kα, and Al kα; and Orthoclase (OR-1) for K kα. The counting time was 30 seconds for all elements. The off-peak counting time was 30 seconds for all elements. The off-peak correction method was Linear for all elements. Unknown and standard intensities were corrected for dead time. Standard intensities were corrected for standard drift over time. Results, other than profiles, are the average of 3 points and detection limits ranged from 0.008 weight percent for Al kα to 0.009 weight percent for Mg k to 0.013 weight percent for Si k to 0.022 weight percent for Ti kα and to 0.034 weight percent for Mn kα. Profiles are equally spaced single-point analyses across a grain from rim to rim. Analytical sensitivity (at the 99% confidence level) ranged from 0.184 percent relative for Si kα to 0.266 percent relative for Ca k to 2.895 percent relative for Cr k to 29.805 percent relative for Mn k and to 149.516 percent relative for Ti kα. Oxygen was calculated by cation stoichiometry and included in the matrix correction. The matrix correction method was ZAF or Phi-Rho-Z Calculations and the mass absorption coefficients dataset was LINEMU Henke (LBL, 1985) < 10KeV / CITZMU > 10KeV. The ZAF or Phi-Rho-Z algorithm utilized was Armstrong/Love Scott (default).

**References:**

1. Van Sprang HA. 2000. Fundamental Parameter Methods in XRF Spectroscopy, copyright © JCPDS-International Centre for Diffraction Data 2000. *Advances in X-ray Analysis*, **42**, 1-10.

2. Reynolds RC Jr. 1963. Matrix corrections in trace element analysis by X-ray fluorescence: Estimation of the mass absorption coefficient by Compton scattering. *Am. Min.* **48**, 1133-1143. (http://www.minsocam.org/ammin/AM48/AM48_1133.pdf)

3. Greenfield S. 1983. Inductively coupled plasma-atomic emission spectroscopy (ICP-AES) with flow injection analysis (FIA), *Spectrochimica Acta Part B: Atomic Spectroscopy* **38**(1), 93-105. (doi.org/10.1016/0584-8547(83)80106-2)

4. Ryan JG *et al.* 2017. Application of a handheld X-ray fluorescence spectrometer for real-time, high-density quantitative analysis of drilled igneous rocks and sediments during IODP Expedition 352. *Chem. Geol.* **451**, 55–66. (doi.org/10.1016/j.chemgeo.2017.01.007)

**Supplementary Figure 1.** Phylogenetic tree showing relationships of DSA OTU and ESA OTU phylotypes from South Chamorro Seamount showing lineages within the *Lokiarchaeota* (formerly Marine Benthic Group B), *Thaumarchaeota* (formerly Marine Group I), and *Crenarchaeota* as determined by maximum likelihood analysis of aligned SSU rDNA sequences. Numbers at nodes represent bootstrap values based on 500 bootstrap resamplings. The outgroup is represented by bacterium *Aquifex pyrophilus*. The scale bar represents 0.10 fixed mutations per nucleotide position. Bootstrap values are shown for frequencies at or above a threshold of 50% (adapted from Curtis *et al.*, 2013, reference [14] in main text).
